# Factors Affecting Intracellular Delivery and Release of Hydrophilic Versus Hydrophobic Cargo from Mesoporous Silica Nanoparticles on 2D and 3D Cell Cultures

**DOI:** 10.3390/pharmaceutics10040237

**Published:** 2018-11-17

**Authors:** Diti Desai, Malin Åkerfelt, Neeraj Prabhakar, Mervi Toriseva, Tuomas Näreoja, Jixi Zhang, Matthias Nees, Jessica M. Rosenholm

**Affiliations:** 1 Pharmaceutical Sciences Laboratory, Faculty of Science and Engineering, Åbo Akademi University, 20521 Turku, Finland; diti.desai@gmail.com (D.D.); nprabhak@abo.fi (N.P.); 2 Institute of Biomedicine, University of Turku, 20520 Turku, Finland; malake@utu.fi (M.Å.); mertor@utu.fi (M.T.); matthias.nees@utu.fi (M.N.); 3 Cell Biology, Faculty of Science and Engineering, Åbo Akademi University, 20521 Turku, Finland; 4 Department of Laboratory Medicine, Division of Pathology, Karolinska Institute, 14186 Stockholm, Sweden; tuomas.nareoja@ki.se; 5 College of Bioengineering, Chongqing University, Chongqing 400044, China; jixizhang@cqu.edu.cn

**Keywords:** intracellular delivery, mesoporous silica nanoparticles, cargo hydrophilicity, cargo hydrophobicity, cancer cells, cell labeling, organotypic 3D culture, live-cell imaging

## Abstract

Intracellular drug delivery by mesoporous silica nanoparticles (MSNs) carrying hydrophilic and hydrophobic fluorophores as model drug cargo is demonstrated on 2D cellular and 3D tumor organoid level. Two different MSN designs, chosen on the basis of the characteristics of the loaded cargo, were used: MSNs with a surface-grown poly(ethylene imine), PEI, coating only for hydrophobic cargo and MSNs with lipid bilayers covalently coupled to the PEI layer as a diffusion barrier for hydrophilic cargo. First, the effect of hydrophobicity corresponding to loading degree (hydrophobic cargo) as well as surface charge (hydrophilic cargo) on intracellular drug release was studied on the cellular level. All incorporated agents were able to release to varying degrees from the endosomes into the cytoplasm in a loading degree (hydrophobic) or surface charge (hydrophilic) dependent manner as detected by live cell imaging. When administered to organotypic 3D tumor models, the hydrophilic versus hydrophobic cargo-carrying MSNs showed remarkable differences in labeling efficiency, which in this case also corresponds to drug delivery efficacy in 3D. The obtained results could thus indicate design aspects to be taken into account for the development of efficacious intracellular drug delivery systems, especially in the translation from standard 2D culture to more biologically relevant organotypic 3D cultures.

## 1. Introduction

Recently, a diverse range of engineered nanomaterials have been developed and widely applied in the industrial, environmental, and biomedical fields [1,2,3,4]. Mesoporous silica nanoparticles (MSNs) have been regarded as especially promising materials for biomedical and pharmaceutical applications, above all drug delivery, owing to their unique surface characteristics, high surface area, large pore volume and good biocompatibility [5,6,7,8,9]. Consequently, bio-functionalized MSNs have been widely applied for controlled drug delivery in targeted cancer therapy [10,11,12,13,14]. Nonetheless, efficacious application of these nanoparticles in tumor diagnosis and therapy requires them to efficiently internalize in tumor cells and tissue, which obviously is essential for the nanoparticles to successfully execute their diagnostic or therapeutic functions in vivo [15]. Therefore, studies consisting of interactions of MSNs with cells, such as the mechanism of cellular uptake and the intracellular trafficking pathway of MSNs within cells, are essential [16]. To date, there have been several studies reported to illustrate the intracellular transport pathway of nanoparticles in cells [17,18,19]. However, it is well known that the intracellular trafficking and uptake mechanism of nanoparticles strongly depends on the size, shape, charge, and surface composition of the nanomaterial as well as the type of cells or cell line and growth conditions; making it challenging to draw any general conclusions. Traditional drugs i.e., small molecules enter the cell predominantly via passive diffusion or active transport, while nanoparticles enter into cells mainly via energy-dependent mechanisms, viz. endocytosis. Endocytosis constitutes de novo production of internal membranes from the plasma membrane lipid bilayer. Thus, plasma membrane lipids and integral proteins and extracellular fluid become fully internalized into the cell [20]. Endocytosis has been deemed the major route for the transport of nanomaterials across the membrane, and is generally classified into phagocytosis and non-phagocytic endocytosis. Phagocytosis includes uptake of larger particles and is one of the key mechanisms by which particles are taken up by macrophages. Non-phagocytic endocytosis includes clathrin-dependent endocytosis, caveolae-dependent endocytosis, macropinocytosis, and clathrin- and caveolae-independent endocytosis. Different endocytosis pathways vary in the vesicle size, proteins involved in the vesicle formation, and the cell type in which they are found. After internalization, the intracellular fate of nanoparticles is dependent on the endocytosis pathway. The endocytotic internalization and appropriate intracellular trafficking is thus a perquisite for effective performance of nano-drug delivery systems.

We have previously studied the effect of surface characteristics on the route of cellular uptake of MSNs [21], and were able to show intracellular release of hydrophobic cargo taking place real-time in live cancer cells [22]. This property has been addressed to the surface-coated poly (ethylene imine) or PEI layer. PEI has been claimed to promote endosomal escape via its unique “proton-sponge” or “endosome buffering” effect, thus destabilizing lysosomal membranes and promoting cargo release into the cytoplasm [23]. This effect has been established previously for PEI-integrated MSNs, but neither the time-frame and kinetics for the release nor the effect of loading degree on uptake and drug release has been pinpointed to date. In an attempt to address this issue, we synthesized fluorescently labeled MSNs with a surface-grown hyperbranched PEI coating to be able to track the mesoporous carrier intracellularly [24]. Additionally, to locate and trace the cargo release microscopically, the MSNs were loaded with a hydrophobic fluorescent dye with a different wavelength emission (DiD or DiI) to be able to distinguish between the cargo and nanocarrier trafficking. To further study the differences between the release of hydrophobic versus hydrophilic cargo, calcein was loaded into the MSNs as a general model for hydrophilic drug molecules. For efficient capping and delivery of hydrophilic cargo, a second coating is required to efficiently retain the cargo within the nanocarrier. In this case, lipid bilayer (LB) tethered nanocomposites (MSN@tLB) were synthesized by covalent tethering of MSN as a core with a lipid bilayer shell via the PEI coating as a cushioning layer [25]. The inner leaflet of the lipid bilayer was composed of DOPE (1,2-dioleoyl-sn-glycero-3-phosphoethanolamine) lipids, whereas, to further analyze the effect of surface charge on endosomal escape, the outer leaflet of the lipid bilayers was varied: DOTAP (1-palmitoyl-2-oleoyl-sn-glycero-3-phospho-(1′-rac-glycerol)) cationic lipid, DOPC (1,2-dioleoyl-sn-glycero-3-phosphocholine) zwitterionic lipid, or POPG (1-palmitoyl-2-oleoyl-sn-glycero-3-phospho-(1′-rac-glycerol)) anionic lipid, were used. MDA-MB-231 cells transfected with Enhanced Green Fluorescent Protein (EGFP) tagged small RabGTPases [26], either as markers for early endosomes (EEA1) or late endosomes (Rab 7) were employed to follow endosomal trafficking and release using live cell microscopy. The presented hybrid nanocarrier systems exhibits a strong capacity to retain both hydrophilic and hydrophobic agents with subsequent specific release into the endosomal compartment. Furthermore, the incorporated agents are also shown to be able to escape from the endosomes into the cytoplasm in a hydrophobicity and/or surface charge dependent manner.

As a final proof-of-concept demonstration, one MSN carrying hydrophobic and one hydrophilic cargo were incubated with organotypic 3D cultures of tumor cells in extracellular matrix (ECM) to gain insight into the capability of MSNs to serve as drug carriers in a more biologically relevant setting [27,28,29]. Tumor cell biology is strongly influenced by the microenvironment. This requires physiologically relevant cell- and tissue models that reflect the effects of the extracellular matrix (ECM), allow cell-matrix interactions, and recapitulate the tissue architecture of solid cancers. Organotypic 3D cell cultures are embedded in ECM preparations like Matrigel and collagen, which allows organoids e.g., to differentiate, polarize and invade. It is widely accepted that 3D organoids reflect in vivo growth and differentiation of epithelial tumor cells more reliably and provide better readouts for drug testing [30,31]. For organotypic cultures, the MSNs used were non-fluorescent; i.e., the detected fluorescence signal distribution would directly correspond to the cargo (hydrophobic DiI or hydrophilic calcein) delivery capability. For hydrophilic cargo-carrying MSN@tLB, parts of the outer LB were further substituted with DiI in an attempt to pinpoint the disassembly of the LB. Here, only diffuse fluorescent signals originating from calcein and DiI were detected with both tested incubation time-points (incubation prior to seeding cells into 3D culture i.e., Matrigel, or incubation at day 3 of 3D culturing). Especially when organoids were incubated directly in the 3D cultures with MSN@tLB, the staining intensity remained very low. However, for hydrophobic cargo-carrying MSNs, the timepoint for incubation of cells with loaded MSNs seemed to be decisive for labeling (=cargo delivery) efficacy. Especially when unlabeled tumor cells were first transferred into 3D matrix in which they formed organoids, the uptake of DiI-loaded PEI-MSNs was much more rapid, effective and long-lasting; which are sought-for traits in the design of MSNs for delivery of bioactives to tumors.

## 2. Materials and Methods

### 2.1. Synthesis and Functionalization of Mesoporous Silica Nanoparticles (MSN)

The starting particles (MSN-NH_2_) were prepared by co-condensation procedure according to a previously reported recipe by us [25]. Initially, a CTAB (0.45 g) solution was prepared in a mixture of water (150 mL) and ethylene glycol (30 mL) at 70 °C in a round bottom flask. Once a clear solution was obtained, decane (2.1 mL) was subsequently added to the mixture. Further, 1,3,5-trimethylbenzene (TMB, 0.51 mL) was added into the mixture after 0.5 h and stirring continued for another 1.5 h to homogenize the solution. Then, ammonium hydroxide (30 wt %, 2.5 mL) was introduced as catalyst, and TEOS (1.5 mL) and APTES (0.3 mL) was added successively to initiate the reaction. To obtain fluorescent MSNs, tetramethylrhodamine (TRITC, 1 mg/mL in ethanol) was pre-reacted with 10 μL APTES at room temperature for 2 h, and added to the reaction mixture. The reaction was continued for 3 h at 70 °C. The molar ratio of the reagents was 1 TEOS: 0.19 APTES: 0.18 CTAB: 0.55 TMB: 1.6 decane: 5.9 NH_3_: 88.5 ethylene glycol: 1249 H_2_O. Subsequently, heating was stopped and the as-synthesized colloidal suspension was then aged at 70 °C without stirring. After the suspension was cooled to room temperature, particles were separated by centrifugation and washed with ethanol. The template was removed by ion exchange method. The purified nanoparticles were redispersed in ammonium nitrate solution (3 mg/mL in ethanol), and then the mixture was stirred at 60 °C for 30 min. The procedure was repeated three times to completely remove the surfactants. The final product was suspended in ethanol for further use.

PEI functionalized MSNs were synthesized by acid-catalyzed hyperbranching surface polymerization of aziridine according to our previous protocols [32,33]. The MSN-NH_2_ particles were centrifuged, washed with toluene two times. Then the particles were dispersed in toluene (20 mL) and were subsequently subjected to argon atmosphere. Catalytic amount of acetic acid was added under stirring, after which aziridine was added in an amount of 0.5 mL per gram of particles. The suspension was stirred under argon atmosphere overnight at 70 °C, centrifuged, washed with toluene, and dispersed in ethanol for further use.

### 2.2. Loading of DiD

To load DiD (1,1′-Dioctadecyl-3,3,3′,3′-tetramethylindodicarbocyanine, Sigma-Aldrich, St. Louis, MO, USA) into the pores of the MSNs, 10 mg of particles were dispersed in cyclohexane to which desired amounts of DiD (0.1% *w*/*w*, 0.5% *w*/*w* and 1.0% *w*/*w*) was added. The resultant loading suspension was stirred overnight. Subsequently, any loosely adsorbed DiD on the particle surface was removed by washing extensively with cyclohexane. The DiD loaded MSNs were then dried in vacuum. Loading of the structural analogue DiI (1,1′-Dioctadecyl-3,3,3′,3′-tetramethylindocarbocyanine) for the organoid studies was conducted the same way.

### 2.3. Loading of Calcein

Loading of Calcein into MSN-PEI was performed by soaking particles in a solution of Calcein in MES (4-Morpholineethanesulfonic acid) buffer (pH 5.0). Subsequently, the mixture was homogenized by continuous rotation at room temperature for 4 h. Then, the Calcein-loaded MSN-PEI particles were separated by centrifugation and the supernatant liquid was collected. The amount of Calcein adsorbed by MSN-PEI was calculated using the UV absorbance before and after the adsorption at a wavelength of 500 nm using a UV-Vis Spectrophotometer (NanoDrop 2000c, Thermo Fisher Scientific Inc., Waltham, MA, USA).

### 2.4. Lipid Bilayer Coating

The lipid bilayer-coated MSNs were prepared by our previously reported method with minor modifications [25]. First, the DOPE (1,2-dioleoyl-sn-glycero-3-phosphoethanolamine) solution in chloroform (0.3 mL, 4 mg/mL) was mixed with DSC solution in anhydrous DMF (0.128 mL, 4 mg/mL). Then *N*,*N*′-dimethylaminopyridine (DMAP, 2 mg/mL in anhydrous DMF, 0.122 mL) was subsequently introduced to the mixture in the presence of activated molecular sieves. The reaction was allowed to proceed for 4 h at room temperature. Then, PEI-MSNs (2 mg) were introduced to the modified DOPE lipid solution to accomplish the crosslinking. The coupling reaction was carried out at room temperature for 4 h. After that, the obtained particles DOPE@PEI-MSNs were separated by centrifugation and resuspended in 0.05 mL chloroform. Subsequently, second lipid solution in chloroform; 1-palmitoyl-2-oleoyl-sn-glycero-3-phospho-(1′-rac-glycerol) sodium salt/1,2-dioleoyl-sn-glycero-3-phosphocholine/*N*-[1-(2,3-Dioleoyloxy)propyl]-*N*,*N*,*N*-trimethylammonium chloride (POPG/DOPC/DOTAP lipid; 0.05 mL, 6 mg/mL) was added to above solution and mixed properly. Then 0.5 mL DMSO was added drop-wise to the mixture. The mixture was mixed and sonicated, and then chloroform was removed completely by vaporization under vacuum at 30 °C for few hours. Afterward, 10 mL of deionized water was added to the suspension with application of sonication. Particles were collected by centrifugation at 6000 rpm for 15 min and resuspended in 2 mL deionized water.

### 2.5. Cell Lines and Culture Conditions

MDA-MB-231 (human breast adenocarcinoma) and LNCaP cells were obtained from American Type Culture Collection (ATCC). MDA-MB-231 cells were cultured in Dulbecco’s modified Eagle’s medium (DMEM) supplemented with 10% fetal bovine serum, 2 mM l-glutamine, and 1% penicillin-streptomycin (*v*/*v*). LNCaP cells were propagated in RPMI-1640 (Sigma-Aldrich), supplemented with 10% FBS, 1% penicillin/streptomycin and 1% l-glutamine. 

### 2.6. Hydrophobic Drug Release

To evaluate intracellular trafficking and amount of drug released from the MSNs with different loading degrees, the empty TRITC-MSNs and DiD loaded TRITC-MSNs were incubated with MDA-MB-231 cells transfected with EGFP-fusions of early endosome marker (EEA1) and late endosome marker (Rab7) [26]. After 24, 48 and 72 h incubation cells were washed with phosphate buffer saline (PBS) and fixed with 4% PFA (paraformaldehyde). The cells were mounted on a cover slip using ProLong Gold antifade reagent (Invitrogen, Thermo Fisher Scientific, Waltham, MA, USA). Subsequently, cells were imaged with confocal microscopy (Leica TCS STED microscope, Wetzlar, Germany). To image cells transfected with EGFP-EEA1/Rab7, TRITC-MSNs and DiD separately, subsequent imaging using laser excitation at 488 nm, 540 nm and 645 nm was used and to decrease overlap between the signals, narrow emission ranges 500–530 nm, 570–600 nm and 660–680 nm were selected. To quantitate amount of the dye release, fluorescent signal from the green channel and TRITC channel was subtracted from DiD channel. Fluorescent intensities were quantified using ImageJ software. Statistical data analysis was performed using GraphPad Prism 8. 

### 2.7. Hydrophilic Drug Release Live Cell Imaging

To evaluate intracellular trafficking, POPG-, DOPC- and DOTAP-DOPE conjugated PEI-MSNs were incubated with MDA-MB-231 cells. The microscopy images of the cells were taken with Leica TCS STED microscope.

### 2.8. Confluency Measurements

The confluency was measured using the real time live cell imaging device Incucyte Zoom (Essen Biosciences, Ltd., Welwyn Garden City, Hertfordshire, UK). The cell culture confluency was measured every hour for all conditions. For comparisons, the confluency values at the starting confluency was normalized to 1, and relative confluency values were presented.

### 2.9. Organotypic 3D Cell Culture

Organotypic 3D cultures were performed as described previously [34]. MSNs were added to the cells either in 2D, before the cells were transferred into Matrigel-based ECM to form 3D culture or while the cell organoids had already started to evolve in 3D cultures.

### 2.10. Addition of MSNs to Cell Culture

PEI-MSNs and MSN@tLB were used at two different concentrations, 10 µg/mL and 30 ug/mL, diluted in RPMI medium supplemented with 10% FBS, 1% penicillin/streptomycin and 1% l-glutamine. MSNs were sonicated 5 min at 37 °C, prior to their addition to the cell culture (2D or 3D). In 2D cultures, the medium was changed after 24 h and cells were subsequently transferred to 3D. When MSNs were added directly into 3D culture, the medium was changed after 48 h.

### 2.11. Live Cell Imaging of Organotypic 3D Cultures

At the experimental end-points, confocal images were taken using a Zeiss spinning disk confocal unit 200 M with 5×, 10× and 20× objectives. Intensity projections were created and background noise was removed by normalization with the SlideBook software (Intelligent Imaging Innovations 3i Inc., Denver, CO, USA).

## 3. Results

**Figure 1:**
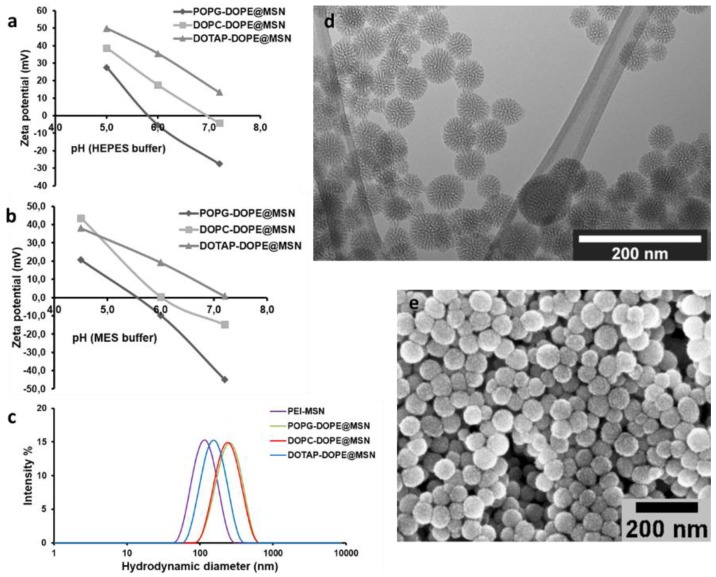
Characterization of mesoporous silica nanoparticles (MSNs). (**a**) Zeta potential values in HEPES buffer and (**b**) MES (4-Morpholineethanesulfonic acid) buffer solutions at different pHs; (**c**) dynamic light scattering (DLS) curves of the MSNs under study; (**d**) TEM and (**e**) SEM images of the unmodified MSNs.

Fluorescently labeled MSNs with a surface-grown PEI layer (for hydrophobic cargo) and MSN@tLB with lipid bilayers covalently tethered to the PEI layer (for hydrophilic cargo) were synthesized and characterized in accordance with our previous protocols [25,35]. Scanning and transmission electron microscopy (SEM, TEM) revealed porous, spherical particles with a sub-100 nm particle size (see Figure 1). Dynamic light scattering (DLS) and zeta potential measurements were also conducted to assure success of surface coating, dispersability after surface functionalization and coating, as well as to study the net surface charge changes of the MSN@tLB under different pH conditions corresponding to the acidic conditions in intrracellular compartments.

### 3.1. Intracellular Uptake and Localization of Non-Loaded PEI-MSNs

**Figure 2:**
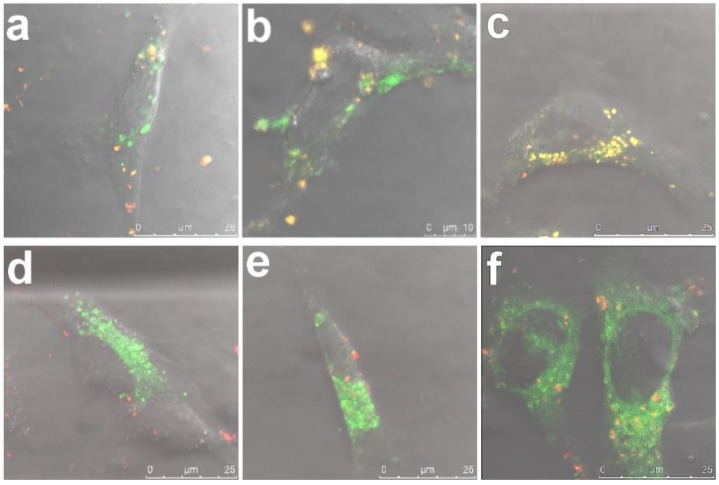
The temporal intracellular trafficking of PEI-MSNs with early endosomes and late endosomes: (**a**–**c**) Localization of PEI-MSNs (red) with GFP-tagged early endosomal antigen1 (green) at 24 h, 48 h and 72 h, respectively. (**d**–**f**) Localization of PEI-MSNs (red) with GFP-tagged late endosomal marker Rab7 (green) at 24 h, 48 h and 72 h, respectively.

The temporal intracellular trafficking by means of compartmentalization with early endosomes (EEA1) and late endosomes (Rab7) was first studied for empty (non-loaded) PEI-MSNs (Figure 2). We first investigated the compartmentalization of PEI-MSNs with an EGFP-fusion of early endosomal antigen-1 (EEA1) as a marker for recently internalized particles via the endosomal pathway [26]. In Figure 2a–c, the PEI-MSNs were observed to be localized with EEA1, thus suggesting that PEI-MSNs were effectively internalized in the cancer cells over the studied 24–72 h time points. There is a gradual increase in colocalization of PEI-MSNs with EEA1 over time, signified by the increased portion of yellow signal (=colocalization). This could be implied as increased cellular uptake with increasing time, which may therefore be actively taking place still at 72 h. In Figure 2c, at the 72 h time point, the majority of PEI-MSNs were seen to be co-localized with EEA1.

In an endosomal internalization pathway, the endosomes gradually mature from high pH (6.5) vesicles (early endosomes) to lower pH (5.5) vesicles (late endosomes) and finally fuse to a low pH (4.5) lysosome [36]. The main function of the lysosome is to degrade material that has not been deemed suitable to be imported into the cytoplasm, due to being unnecessary or harmful for normal cellular functions. The gradual degradation of such materials is due to its low pH and degrading enzymes in the lysosomes, such as hydrolases. Consequently, many drug molecules are likely to be inactivated/degraded in the acidic lysosomal compartment. Therefore, it would be desirable for most active compounds if they could be released and escape early stage carrier vesicles prior to being trafficked to the late lysosome. Thus, we selected EGFP-Rab7, a marker for late endosomal vesicles [26], to investigate intracellular trafficking of PEI-MSNs as delivery system. In Figure 2d–f, PEI-MSNs are seen increasingly co-localized with Rab7 over time; however, to a lesser degree than with early endosomes. In line with the observation above, we interpret this as a sign that the cellular uptake of MSNs can continue over a long period of time and that, at 72 h after incubation, the majority of MSNs are still compartmentalized in endosomes.

### 3.2. Factors Affecting Intracellular Release on Cellular Level

**Figure 3:**
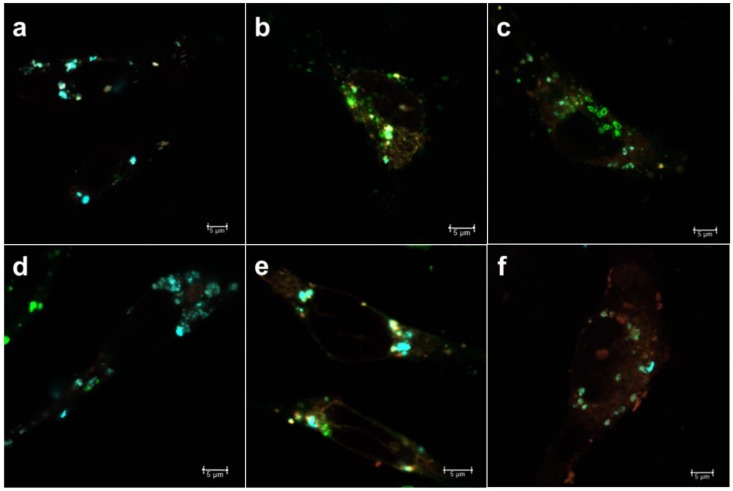
MSNs (cyan) loaded with fluorescent dye (DiD) (red) and endosomal markers (green) (**a**–**c**) early endosomal antigen-1 (EEA1) (0.1, 0.5, 1.0 wt % loading of DiD, respectively) and (**d**–**f**) Rab7 (0.1, 0.5, 1.0 wt % loading of DiD, respectively) at 24 h.

**Figure 4:**
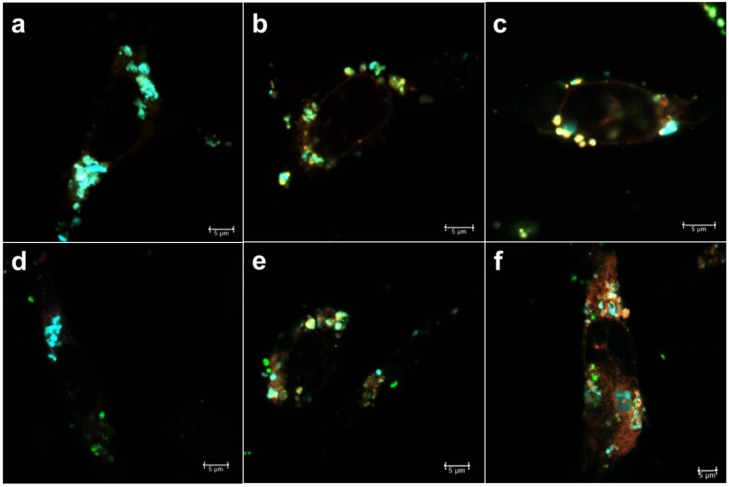
MSNs (cyan) loaded with DiD (red) and endosomal markers (green) (**a**–**c**) EEA1 (0.1, 0.5, 1.0 wt % loading of DiD, respectively) and (**d**–**f**) Rab7 (0.1, 0.5, 1.0 wt % loading of DiD, respectively) at 48 h.

**Figure 5:**
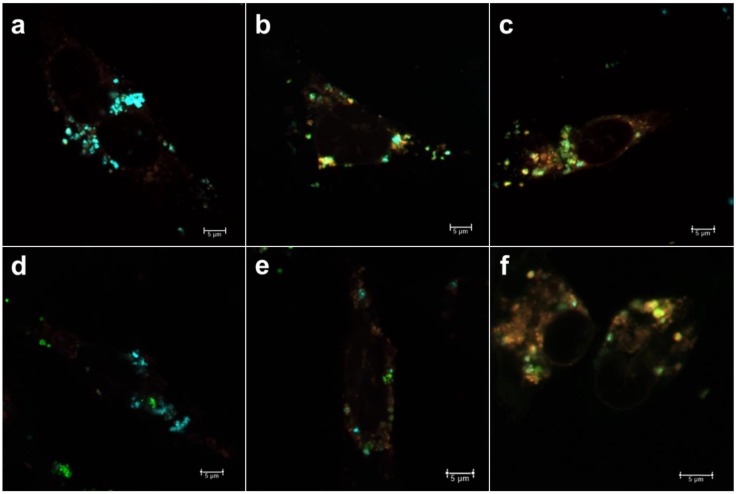
MSNs (cyan) loaded with DiD (red) and endosomal markers (green) (**a**–**c**) EEA1 (0.1, 0.5, 1.0 wt % loading of DiD, respectively) and (**d**–**f**) Rab7 (0.1, 0.5, 1.0 wt % loading of DiD, respectively) at 72 h.

**Figure 6:**
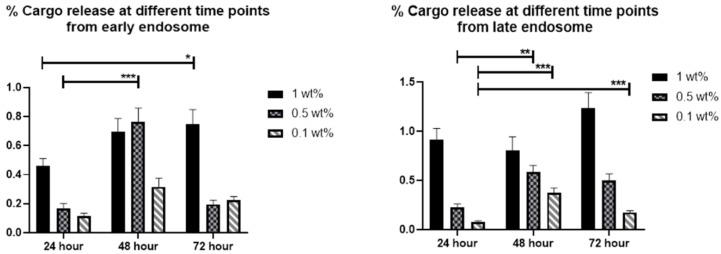
Relative amount of non-localized cargo signal with the endosomal marker signal over time, representing the effect of DiD loading amount on the release from early/late endosomes (* *p* ≤ 0.05; ** *p* ≤ 0.01; *** *p* ≤ 0.001).

For the loaded compounds to be able to reach the cytoplasm, either the loaded carrier should escape from the intracellular compartments or, alternatively, the release of cargo can take place in the intracellular compartments, after which the cargo molecules themselves need to escape to the cytoplasm before reaching the lysosomes. For larger (approx. 300 nm) MSNs, we have observed that the endosomal escape primarily seem to take place for cargo molecules after release from the carrier [22,37]. However, for sub-100 nm particles we have not studied the mechanism of intracellular release and endosomal escape. Thus, to be able to trace the intracellular cargo release and distinguish this signal from the nanocarriers themselves, the MSNs were further loaded with a fluorescent dye (DiD) as hydrophobic model drug cargo at different loading degrees (0.1, 0.5 and 1 wt %), whereas calcein (10 wt %) was used as a model for hydrophilic drug molecules. The reason for choosing such low loading degrees for the hydrophobic cargo was that we have previously studied higher loading degrees (5–10 wt %) and thereby been able to conclude that the release becomes sustained over several days [22], and we here attempted to pinpoint the release at shorter timepoints. Thus, by separately tracing the nanocarriers’ trafficking vs. that of the cargo, the effect of loading amount on release of hydrophobic cargo was investigated, in tandem to the effect of surface charge/hydrophobicity on the endosomal escape of the cargo. The rationale behind choosing these parameters are owing to the supposed impact of wetting effects in the case of hydrophobic cargo. Namely, a higher amount of hydrophobic cargo loading would lead to an increased hydrophobicity of the overall drug carrier system, which could have implications on the release rate in terms of delayed release [38] with increased loading of hydrophobic agents. Consequently, a higher loading degree could provide a release of cargo over a prolonged period of time [39]. In our earlier studies, we have clearly observed a delayed but efficient intracellular release within a couple of days [37,39] when using hydrophobic dye molecules at a loading degree of 5 wt %, in spite of hardly any release taking place under in vitro conditions in buffer solution only over a time course of one week [22]. This makes it difficult to study release kinetics under standardized conditions, which would in this case be meaningless since the observed release kinetics are not translatable to intracellular conditions. Similar behavior has been observed for hydrophobic drug molecules [12,40]. Therefore, we chose to study the intracellular release by means of compartmentalization with the aid of the above-mentioned endosomal markers EEA1 and Rab7, whereby a lower degree of colocalization corresponds to a higher degree of release from the endosomal compartment (Figure 3, Figure 4, Figure 5 and Figure 6).

From Figure 6, one can discern that for very low loading degrees (0.1 wt %) the release at the first time point (24 h) is low, whereas it increases for the second timepoint (48 h) and decreases again at the last timepoint (72 h). This pattern holds true for both early and late endosomes. A similar pattern is seen for the 0.5 wt % loaded MSNs, but which higher fraction of release at all timepoints and both compartments. This can be understood, as the loaded amount is five times lower for the 0.1 wt % loaded MSN; and small hydrophobic molecules in low amounts can bind very strongly to the MSN matrix to the extent that they may not be released [41]. For the 1 wt % loaded sample, the release in general increases over time. This should come as no surprise, as we have previously been able to follow the fluorescence signal of 5 wt % DiI (structurally analogous to DiD) loaded MSNs in vivo up to one month [39] and monitored the real-time release of DiI dye from endosomes with the aid of fluorescence recovery after photobleaching (FRAP) [22]. Here, we have ascribed the rentention, prolonged/sustained release as well as endosomal escape of dye to the hyperbranched PEI layer on the MSNs. Without this coating, MSNs loaded with (too) high amounts of hydrophobic dye would not be properly dispersible in an aqueous environment [42] nor would endosomal escape most likely be efficient [22]. For the range studied here, it is evident that the loading degree also has an effect on both the relative intracellular release rate as well as on duration and extent.

**Figure 7:**
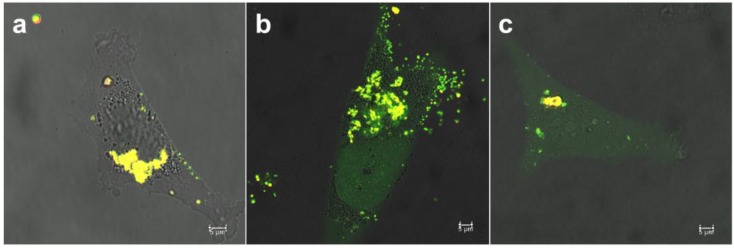
Microscopy images of net surface charge effect on the endosomal escape of calcein loaded in (**a**) POPG-, (**b**) DOPC- and (**c**) DOTAP-DOPE@PEI-MSNs. Surface charge of second lipid in lipid bilayer (POPG—negative charge, DOPC—neutral, and DOTAP—positive charge) influence the drug/calcein release from endosomes, visualized as uniform spreading in water containing cytoplasm compartment. Overlay of bright field and fluorescent signal from TRITC labeled tLB@MSNs (red) and calcein (green).

In our previous studies, we have also been able to pinpoint that the MSN net surface charge can have a profound effect on the resultant intracellular patterning of cargo molecules and subsequent release to the cytoplasm [25,35]. When loaded with hydrophilic cargo molecules, the PEI-MSNs need a diffusion barrier to efficiently retain the loaded molecules in an aqueous environment. For this purpose, we developed a so-called “protocell” [43] type MSN in which the first leaflet is covalently tethered to the cushioning PEI-layer, thereby providing excellent retention of loaded molecules even in the presence of surfactants. Consequently, this presumed stability of the system could be verified also in vivo [35]. As shown in Figure 1, depending on the outer lipid layer, the overall system acquires different net surface charge (zeta potential) whereby charge reversal can even take place at the acidic intracellular conditions as compared to neutral extracellular pH. The effect of the outer lipid layer on the intracellular release of calcein is shown in Figure 7.

The specific endocytosis pathways involved in cellular uptake of nanocarriers can be studied by using inhibitory drugs that specifically interfere with one or the other endocytic pathway [20], whereby the cellular uptake route has shown to be net surface charge dependent [21,35]. In the present case, the intracellular trafficking after uptake was studied as a dependence of the outer lipid layer, which also determines the overall net surface charge (Figure 1a,b). Clearly, the extent of colocalization (yellow) between MSNs and released calcein (green) decreases in the order POPG > DOTAP > DOPC (indicating a reverse efficiency of intracellular release and endosomal escape). This is in accordance with our earlier observations [35] and can most likely be ascribed to the acquired positive charge at intracellular conditions, especially for DOTAP and DOPC coated MSNs (Figure 1a,b) where the positive charge stems from the LB-underlying PEI layer.

### 3.3. Factors Affecting Intracellular Release on Organoid Level

**Figure 8:**
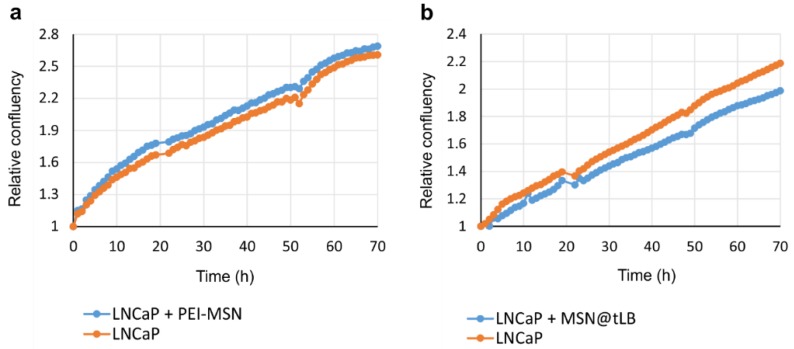
Confluency measurements over time for cells with and without MSNs. The confluence of LNCaP cells unlabeled, labeled with PEI-MSNs (**a**) or labeled with MSN@tLB (**b**), was measured using live cell imaging device Incucyte ZOOM. For comparison between the different conditions, the initial confluency values were normalized to 1, and relative values were presented over time (hours).

Before incubating MSNs with 3D organoids, the growth of tumor cells cultured in 2D was monitored using live cell microscopy to detect if the addition of PEI-MSNs and MSN@tLB affect the cell culture confluency (and thus growth and proliferation) over time, while the cytocompatibility on 2D cell cultures were elucidated already in our earlier studies. No major differences in proliferation were observed between the cells incubated with MSNs and tumor cells without added MSNs (Figure 8).

**Figure 9:**
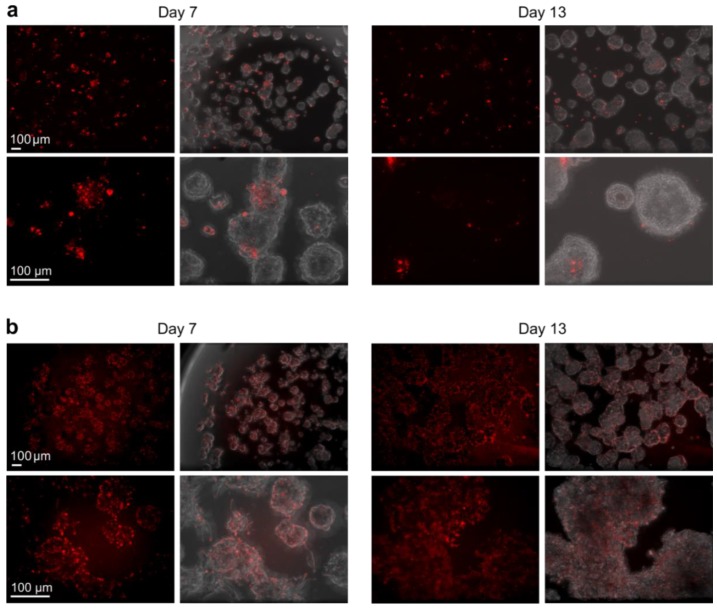
Labeling efficiency of DiI dye as delivered by PEI-MSNs in LNCaP 3D organoids. Comparison of the labeling efficiency of DiI loaded in PEI-MSNs in LNCaP cells when added to cells in 2D culture and then transferred into 3D culture (**a**), or when added directly into the 3D culture, at day 3 (**b**), in a concentration of 10 µg/mL. Confocal images show the red fluorescent cargo (DiI), which has been monitored at two different time points (day 7 and 13 of 3D culture) inside the tumor organoids (bright field).

To investigate the MSN-mediated labeling efficiency in a more biologically relevant setting, which better recapitulate the in vivo conditions observed in cancer patients, organotypic 3D cultures were utilized. In the 3D cultures, LNCaP prostate cancer cells were embedded in physiologically relevant ECM (laminin-rich Matrigel), which allows the cells to grow, differentiate and potentially invade. MSNs were added either to tumor cells cultured in standard 2D conditions that were subsequently transferred into organotypic 3D culture, or directly into already pre-established 3D cultures at different time points. The delivery/labeling efficiency of DiI (structural analogue to DiD) loaded in PEI-MSNs and calcein in MSN@tLB (PEI-MSN@DOPC-DOPE) were examined. A small fraction of the outer lipid layer was further substituted with DiI, to potentially reveal something about the disassembly of the LB [25] in 3D. When PEI-MSNs were added to the cells first in 2D, and these cells were later transferred to 3D cultures, intracellular labeling efficiency of loaded dye was only poor to intermediate. Especially after an extended time of 3D culture, the fluorescent signal was clearly diminished (Figure 9a). Interestingly, the labeling efficiency of PEI-MSN was significantly increased when particles were added directly into the matrix, containing the embedded tumor organoids (Figure 9b). In this setting, the dye signal was observed quite homogeneously inside the organoids. Even after longer periods of 3D culturing, the fluorescent signal from the cargo remained strong and homogeneous in the tumor organoids (Figure 9b). This could indicate an active mode of uptake at least into organoids, if not into tumor cells within these organoids. This is possibly due to the active turnover of the matrix by such tumor organoids that could support active particle association and enhanced uptake. 

**Figure 10:**
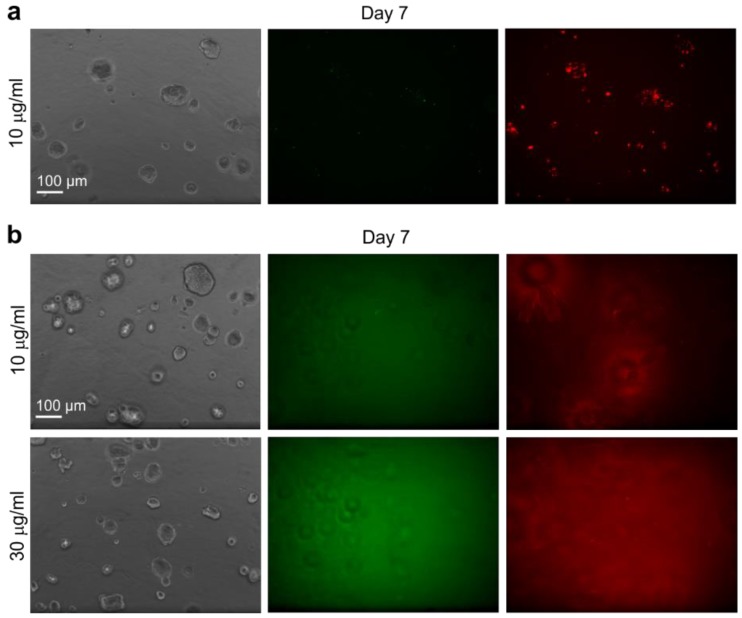
Labeling efficiency of hydrophilic calcein and hydrophibic DiI dyes as delivered by MSN@tLB in LNCaP 3D organoids. Comparison of the labeling efficiency of cargo loaded into MSN@tLB in LNCaP cells when they are added to cells in 2D culture prior to transferring cells into 3D culture (**a**), or added directly into the 3D culture at day 3 in two concentrations, 10 μg/mL and 30 μg/mL (**b**). Confocal images shows tumor organoids (phase contrast) and a diffuse staining of green cargo that is a model for hydrophilic drug molecules (calcein), and the red fluorescent cargo that is modeling a hydrophobic drug cargo (DiI) inserted into the lipid bilayer coating. The imaging was conducted at day 7.

When the calcein-loaded MSN@tLB were added to cells first in 2D and these cells were transferred to 3D cultures, the labeling efficiency was not efficient, probably due to poor particle uptake (Figure 10a). Only small clusters of MSN@tLB were visible inside the organoids, similar to what was observed with the PEI-MSNs that were added in 2D and later transferred to 3D culture (Figure 9a). However, when MSN@tLB were added directly into the organotypic 3D cultures, the particles were immobilized and retained in the ECM. In this setting, it seems no particles were taken up by cells nor incorporated into the organoids (Figure 10b). There was no difference between visualization of hydrophobic (DiI) or hydrophilic (calcein) model cargo, only an unspecific, blurry fluorescent signal was detected while imaging the 3D cultures (Figure 10b). Taken together, our results suggest that PEI-MSNs without a lipid bilayer coating are most efficiently taken up and released its cargo in the tumor organoids when distributed directly into the ECM.

## 4. Discussion

From the confocal microscopy images of individual cells, we have observed that both hydrophobic and hydrophilic cargo can be efficiently released intracellularly from MSNs. Especially for the hydrophobic cargo, loading degree plays a key role for its release efficiency and kinetics inside the cells. For the release of hydrophilic cargo, the surface charge of the second lipid bilayer plays a significant role for the endosomal escape of the cargo.

### 4.1. Effect of Surface Charge on Intracellular Trafficking and Release

It has been recognized that the cytomembrane possess negative charge, therefore, cationic/positive charged nanoparticles (NPs) may show a strong electrostatic interaction with the cell surface, which can result in a rapid cellular entry. Cationic NPs can also escape from endosomes after cellular internalization and exhibit perinuclear/cytoplasmic localization supposedly because of the ‘proton-sponge’ effect. Neutral NPs at physiological pH may interact with the cell surface with the help of hydrogen bonds and hydrophobic interactions. Negatively charged NPs are most probably endocytosed through the interaction with the positive site of the proteins in the cell membrane, and they can be highly captured by cells because of their repulsive interactions with the negatively charged cell surface [44]. Surface charge of a nanoparticle can be a crucial parameter to determine which trafficking pathway will be dominant. Therefore, we have in this study used three differently charged lipids for the formation of the second layer of the outer lipid bilayer assembly tethered onto MSNs. In our earlier study, no difference in extent of uptake or internalization pathway was observed for any of the three MSN@tLB, but for the plain PEI-MSN (without LB) the behavior was different, pointing to that another route of uptake may have been activated [35]. The reason for more efficient intracellular release displayed by the DOPC-terminated LB coating, despite having almost identical net surface charge to the DOTAP-terminated one under the studied conditions (acidic pH) may be related to its zwitterionic nature. Zwitterionic MSN coatings have been shown to adsorb a significantly lesser extent of serum proteins onto its surface, whereby the formation of such a ‘protein corona’ could both hamper cellular uptake and/or hinder intracellular release.

### 4.2. Effect of Surface Hydrophobicity on Intracellular Release

Hydrophobic NPs in general have higher affinity for the cell membrane than do hydrophilic NPs, leading to an improvement of cellular uptake efficiency and kinetics [17]. On a cellular level, hydrophilic polymer coatings that are frequently used to prolong the blood circulation time, form a steric barrier to suppress the interaction between the NPs and LB (i.e. membrane) of cells tandem to acting as adsorption barrier against plasma proteins. It has thus been found that the kind of polymer used as coating may contribute to the cellular uptake route selection [17]. For empty but larger (approx. 300 nm) MSNs, no difference in route of uptake was observed as a function of surface functionalization; but the positively (PEI), neutral (PEG) and negatively (succinylated, i.e., COOH–terminated) charged MSNs all internalized via similar endocytosis pathways [21]. In the present case, the highest (1 wt %) loading degree also provided the most efficient intracellular release at the earliest timepoint (24 h) and continued to increase until the latest studied timepoint (72 h) which may very well reflect the hydrophobic characteristics. In accordance with the reasoning above, a more hydrophobic system would lead to more efficient uptake also at early timepoints. When studied in parallel, it has been observed that a greater degree of loaded PEI-MSNs was colocalized with endosomes at 24 h than empty PEI-MSNs, pointing to different routes of internalization owing to the different degrees of hydrophobicity [45]. Nevertheless, for very low loading degrees, the adsorbed compounds may interact so strongly with the MSN matrix that they may not be released until degradation of the MSN occurs. These observations highlight the fact that also the loading degree needs to be optimized depending on the desired type of therapeutic response.

### 4.3. Effect of Surface Coating (PEI vs LB) on the Labeling Efficiency of Loaded Dye in 3D Organoids

The uptake of MSNs by tumor cells or organoids seems to strongly depend on their microenvironment, the characteristics of the MSN surfaces, but notably, also on the type of cell line. In the pre-labeling step for organoid formation, where only small amounts of matrix proteins are produced, the uptake of both lipophilic dye-carrying PEI-MSNs and hydrophilic dye-carrying MSN@tLB appears inefficient. This is in stark contrast to the cellular studies above, where fast-dividing MDA-MB-213 cells was used. For organoid studies, LNCaP cells were preferred since this line readily generates well-differentiated, polarized organoids that resemble genuine tumor “islands” in solid prostate cancers. Such organoids are typically round and contain a basement membrane that separates the tumor structure from the ECM. It is of particular interest if MSNs or in fact any NPs can be actively incorporated into such tissue-like structures in vitro. We have previously observed clearly different extents of MSN uptake in different breast cancer cell lines in 2D [12,46]. In LNCaP cells, the MSNs appear to remain restricted to the cell surface and are not incorporated into organoids, when these cells are later transferred into ECM. In contrast, embedding the dye-loaded PEI-MSNs into the ECM appears to represent an effective way for labeling cancer organoids and the tissue-like structures that are forming under these conditions. The uptake of PEI-MSN particles, but not that of MSN@tLB seems to be highly active and dynamic, probably depending on the dynamic turnover and proteolytic modification of the tumor microenvironment and matrix by tumor organoids.

As noted above, the MSN characteristics may contribute to the intracellular distribution and release on cellular level, but significantly so also on tissue level. In this study, we observed insufficient degree of dye labeling (cargo delivery) in the tumor organoids by calcein delivered by MSN@tLB; albeit almost the same design was successful in suppressing tumor growth in vivo in a murine breast cancer model [35]. Parts of the LB in the MSN@tLB in the in vivo study were replaced by PEG chains conjugated to folic acid (FA) perturbing from the LB surface, which may have provided the crucial difference since it is well-known that PEGylated liposomes are more efficient drug delivery carriers to tumors than non-PEGylated. PEI-coated MSNs have, as in this study also confirmed on 3D organoid models, been shown to work as efficient drug/dye carriers in vivo both for cellular labeling as well as drug delivery purposes [12,39,40]. All in all, it stands clear that after pinpointing the crucial factors for efficient drug delivery on cellar level, the effect of the same parameters (surface charge, hydrophobicity, delivery efficiency) need to be verified also on a more in vivo relevant model to enable rational design of translatable nanocarriers.

## 5. Conclusions

The effect of physical and chemical properties of nanoparticle surfaces plays a significant role in specific interactions with cell surfaces and intracellular components. The presented nanocarrier systems exhibit capacity to retain both hydrophilic and hydrophobic agents with subsequent specific release into the endosomal compartment. The incorporated agents are able to escape from the endosomes into the cytoplasm in a loading degree (hydrophobic cargo) or surface charge (hydrophilic cargo) dependent manner, making the particles promising candidates as versatile carriers for intracellular drug delivery. In 3D tumor organoid models, PEI-MSNs carrying hydrophobic molecules were shown to be able to efficiently deliver its cargo into the cancer cells, regardless of 3D culture conditions, timepoint and organoid differentiation status; whereas MSN@tLB carrying hydrophilic molecules provided insufficient delivery of cargo into the tissue-like structures that are formed in 3D cultures. It is also striking that PEI-MSNs are able to enter organoids despite the prominent formation of rigid basement-membrane-like structures especially by LNCaP organoids, otherwise a formidable boundary for incorporation of either drugs or NPs. One can postulate that the active re-arrangement and turnover of the extracellular matrix, a feature observed routinely in 3D organoid cultures especially with physiologically relevant matrices, may offer an entry point for active penetration of MSNs into multicellular organoids.
